# Association Between Monocyte-to-High-Density Lipoprotein (HDL) Cholesterol Ratio and Proteinuria in Patients With Type 2 Diabetes Mellitus: A Prospective Observational Study

**DOI:** 10.7759/cureus.45783

**Published:** 2023-09-22

**Authors:** Yashilha D, Shini Rubina SK, Nanda Kumar R, Anuba PA

**Affiliations:** 1 Department of General Medicine, Faculty of Medicine and Health Sciences, SRM Institute of Science and Technology, SRM Medical College Hospital and Research Centre, Kattankulathur, IND; 2 Department of Pharmacy Practice, Faculty of Medicine and Health Sciences, SRM Institute of Science and Technology, SRM College of Pharmacy, Kattankulathur, IND

**Keywords:** metabolic syndrome, metabolic disorder, creatinine, albuminuria, diabetic nephropathy, diabetes mellitus

## Abstract

Background

Diabetes mellitus (DM) refers to a group of metabolic disorders that share the phenotype of hyperglycemia. “Diabetic nephropathy (DN)" is a microvascular complication of DM, and it is the leading cause of end-stage renal failure. Increased urinary albumin excretion (UAE) and a decrease in glomerular filtration rate (GFR) are associated with DN along with elevated blood pressure and end-stage renal disease (ESRD). The purpose of this study is to analyze the prognostic significance of the monocyte-to-high-density lipoprotein (HDL) cholesterol ratio (MHR) in DN patients.

Materials and methods

This prospective observational study was carried out over a period of 1.5 years, with patients being followed up for three months. One hundred twenty participants were enrolled and allotted into groups based on the measure of urine albumin-to-creatinine ratio (UACR). The participants were categorized into healthy individuals, normoalbuminuric diabetic patients, microalbuminuric diabetic patients, and macroalbuminuric diabetic patients group. The MHR, neutrophil-to-lymphocyte ratio (NLR), and platelet-to-lymphocyte ratio (PLR) were estimated and compared between the baseline measurements.

Conclusion

The MHR, NLR, and PLR showed a positive correlation with UACR levels which could serve as an inflammatory marker and be used as an inexpensive and accessible prognostic marker in DN patients.

## Introduction

Diabetes mellitus (DM) refers to a group of metabolic disorders that share the phenotype of hyperglycemia. Several types of type 2 DM (T2DM) are caused by a complex interaction of genetics and environmental factors [1]. The prevalence of diabetes is higher in developing countries. Apart from the increased risk for cardiac disease, T2DM raises the incidence of cerebrovascular accidents. T2DM is also the primary cause of "acquired blindness," and it is the cause of 1/4 of cases of end-stage renal disease (ESRD) and almost 1/2 of cases of amputation of the lower limb that is not related to trauma [2]. Diabetic nephropathy (DN) is a microvascular complication of T2DM, and it is the leading cause of end-stage renal failure. DN is chiefly seen in 25-40% of patients with T2DM [3]. Increased urinary albumin excretion (UAE) and a decrease in glomerular filtration rate (GFR) are associated with DN along with elevated blood pressure and ESRD [4-6]. Microalbuminuria is the earliest sign of nephropathy, which is also a definitive marker of cardiovascular morbidity and mortality in T2DM patients [7-9]. DN can be divided into stages depending on UAE values: microalbuminuria and macroalbuminuria [10-11]. Within a span of 10 years, more than 80% of patients with T2DM associated with microalbuminuria progress to clinical nephropathy. Inflammation plays an important role in the development of DN and its progression [12-14]. Prolonged low-grade inflammation was found to be associated with increased UAE. Decreased levels of high-density lipoprotein (HDL) and increased monocytes were found to precipitate atherosclerosis [15-16]. The monocyte-to-HDL cholesterol ratio (MHR) demonstrates oxidative stress and inflammation due to the pro-inflammatory effect of the monocytes and also the anti-oxidant and anti-inflammatory effect of the HDL. MHR has been considered a marker of systemic inflammation and also has been found in recent studies to be associated with coronary artery disease [17]. In this study, the aim is to examine the relationship between the level of UAE, an indicator of DN and MHR.

DN

Historical Facts

As early as 1936, Kimmelstiel and Wilson outlined the pathology of DN through autopsy. Eight cases had the typical DN nodular glomerulosclerosis variety of the disease. Diffuse glomerulosclerosis was distinguished from the nodular forms [18]. Glomerular basement membrane (GBM) thickening and expansion of mesangium was found to be the earliest change in DN demonstrated by electron microscopy and renal biopsy. The epidemiology of DN can be better presumed in T1DM patients. During their lifespan, 25% to 45% of patients develop DN. DN usually develops at around 10 to 15 years from disease onset. The prevalence of DN was found to be lesser in T2DM patients. Fifty percent of T2DM patients developed DN. Fifteen percent of patients with DN showed progression to ESRD after 20 years. Proteinuria may predispose to cardiac disease [19-20]. The exact prevalence of DN is hard to figure out. The development of cardiovascular changes would have anteceded the development of ESRD [21]. Recent studies have depicted that the risk of developing nephropathy is the same for both types of DM. In both types of DM, the onset and progression to ESRD from the time of proteinuria were the same [22].

Natural History of DN for T1DM

Mogensen et al. proposed five stages of kidney involvement in DN [23] as follows:

Stage 1: glomerular hyperfiltration: Even with optimal blood glucose, GFR remains above normal in 25% to 40 % of patients. In this group, compared to controls with normal GFR, the GFR fell more rapidly. This raised GFR is due to the increased glomerular capillary filtration surface. Higher GFR early in the course of the disease makes it more likely to develop DN.

Stage 2: early lesions: Mild thickening of the GBM takes place 18 to 24 months from the start of T1DM. Glycosylation of the basement membrane causes an increase in the filtration of proteins. The negative charge of the basement membrane is reduced which causes increased loss of albumin as it is no longer repelled by the basement membrane [23].

Stage 3: incipient DN (microalbuminuria stage): Microalbuminuria is urine albumin excretion of 20 to 200 g/min per day. It is associated with loss of renal function and poor outcomes and with vascular injury in other organs [22].

Stage 4: clinical nephropathy (macroalbuminuria, decline in GFR): This usually manifests in patients who have diabetes for 15 to 20 years. Without intervention, the GFR in these patients falls at about 1 mL/min/month.

Stage 5: ESRD: This occurs after 20 to 30 years of diabetes. This is seen in 30% to 40% of patients with T1DM. Uremic symptoms and signs are seen at much higher creatinine clearances than non-diabetics. Clinical predictors and risk factors for DN include glycemic control, duration of disease, blood pressure, and GFR [24].

Urine albumin-to-creatinine ratio (UACR)

The level of albumin in urine and the estimated GFR (eGFR) are two main predictors of chronic kidney disease (CKD). The National Kidney Disease Education Program recommended the yearly assessment of levels of urine albumin to observe the extent of kidney damage for all patients with T2DM irrespective of disease duration. Frequent monitoring is required in patients whose clinical status shows changes or for patients who have undergone therapeutic intervention.

Since albumin is the smallest protein, pathologic proteinuria typically begins with the excretion of albumin from the urine. A spot urine sample can be used to estimate the urine albumin level. It is better to find the association between the amount of albumin excretion against its creatinine concentration to compensate for the variations in concentration in urine. Such a comparison is known to be the UACR.

UACR is known as the ratio between the two substances. Urine albumin (expressed as mg/dl) and urine creatinine (expressed as g/dl) are measured values. UACR is used to estimate the 24-hour excretion of urine albumin. UACR is represented as mg/g and approximately measures the amount of excretion of albumin in urine in mg/day. UACR is not affected by urine concentration variation, unlike the dipstick method. The first voided morning urine sample is used for UACR. Twenty-four-hour urine collection or timed specimens are not specifically required. A UACR greater than 30 mg/g suggests the presence of albuminuria, which is a marker of CKD.

Albuminuria is used to diagnose and monitor the progression of kidney disease. The responses to therapy and risk for progression to CKD can be monitored using urine albumin levels. The decrease in the level of urine albumin can be associated with the improvement of cardiovascular and renal outcomes. One of the primitive signs of DN is microalbuminuria; therefore, preferably a sample taken early is used. The patient should not have done heavy exercises 24 hours prior to the test. The test must be repeated three or six months after assessing the microalbuminuria for the first time. Inflammation plays an important role in the development of DN and its progression [12]. Prolonged low-grade inflammation seems to be associated with increased UAE. Decreased levels of HDL and increased monocytes were found to precipitate atherosclerosis [17]. The MHR demonstrates oxidative stress and inflammation due to the pro-inflammatory effect of the monocytes and the anti-oxidant and anti-inflammatory effect of the HDL. MHR is considered a marker of systemic inflammation and has been found in recent studies to be associated with coronary artery disease. In this study, the aim is to examine the relationship between the level of UAE, an indicator of DN and MHR.

## Materials and methods

Study design

After obtaining approval from the Institutional Ethical Committee (1635/IEC/2019), a prospective observational study was conducted among patients with T2DM in SRM Medical College Hospital and Research Centre for a period of 18 months from March 2018 to August 2020. The study was carried out under the Helsinki Declaration and good clinical practice recommendations.

Inclusion and exclusion criteria

This study recruited patients above 18 years old. To find significance in DM patients and prevent confounding effects with hypertensive nephropathy and known CKD patients, patients with systolic blood pressure above 140 mmHg, diastolic blood pressure above 90 mmHg, and eGFR <60 ml/min/1.73m2 were not included in the study. Patients with documented malignancy, acute or chronic infection, inflammatory disease, chronic obstructive pulmonary disease, asthma, hematological disease, coronary artery disease, heart failure, peripheral vascular disease, and cerebrovascular disease and those receiving lipid-lowering drugs were excluded from the study.

Study method

In this prospective observational study, patients seeking hospital during the period of March 2018 to the end of August 2020 were screened for eligibility based on inclusion and exclusion criteria. Based on the inclusion and exclusion criteria, we enrolled 120 patients prospectively. These patients were categorized into four groups: Group 1 includes patients who come to the internal medicine OPD for routine health check-ups and have no documented disease, Group 2 includes patients with normoalbuminuric DM, Group 3 includes microalbuminuric diabetic patients, and Group 4 includes macroalbuminuric diabetic patients.

Measuring tool

All the enrolled patients were screened for spot UACR. The patients whose UACR was less than 30 mg/g were assigned to the normoalbuminuric group. The patients with UACR 30-300 mg/g were assigned to the microalbuminuric group, and the patients with UACR 300 mg/g were assigned to the macroalbuminuric group. The patients with microalbuminuria and those with macroalbuminuria were considered to have DN.

Laboratory investigations

Blood samples were collected from all participants in the morning after 12 hours of fasting. Spot UACR in the urine specimens was taken as the first voided morning sample. HbA1c, hemoglobin, total white blood cell count, serum creatinine, HDL cholesterol, total cholesterol, triglyceride, and C-reactive protein (CRP) were analyzed and compared between the groups. The MHR, NLR, and PLR will be calculated, and the same will be repeated after three months. These findings were analyzed and compared statistically.

Statistical analysis

The collected data were analyzed with SPSS Statistics version 23.0 (IBM Corp. Released 2015. IBM SPSS Statistics for Windows, Version 23.0. Armonk, NY: IBM Corp.). To describe the data descriptive statistics frequency analysis, percentage analysis was used for categorical variables, and the mean and SD were used for continuous variables. To find the significant difference in the multivariate analysis, the one-way ANOVA was used. To find the significance in categorical data, the Chi-square test was used. In both the above statistical tools, the probability value of 0.05 is considered a significant level.

## Results

In this prospective observational study, patients who were eligible for the study were included from the period of March 2018 until the end of August 2020. This study enrolled 120 patients, of which 5.0% were under 40 years and 15.0% were above 60 years. Among enrolled patients, 54.2% were female and 45.8% were male. After obtaining consent, blood samples were collected from enrolled patients, and the parameters were analyzed between the groups using one-way ANOVA. One-way ANOVA revealed a statistically significant difference in eGFR between the groups (F-value=5.832, P=0.001). Results of the one-way ANOVA analysis for hemoglobin showed a significant difference (F-value=5.775, P=0.001). Results of the one-way ANOVA analysis for total counts between the groups had a statistically significant difference (F-value=3.596, P=0.016). Results of the one-way ANOVA analysis for HDL between the groups showed a significant difference (F-value=2.968, P=0.035). Results of the one-way ANOVA analysis for the absolute lymphocyte count between the groups indicate no significant difference. A significant difference was observed in neutrophil count (F-value=5.245, P=0.002), absolute monocyte count (F-value=23.479, P<0.001), platelet count (F-value=9.853, P<0.001), high-sensitivity CRP (hsCRP) (F=31.775, P<0.001), and HbA1c (F-value=94.65, P <0.001). Comparing spot urine ACR among all groups obtained a statistically significant difference (F-value=296.542, P<0.001). Similarly, the NLR value at baseline and after three months showed a statistically significant difference (F-value=16.037, P<0.001). The baseline measure and three-month follow-up measure of the MHR manifested a statistically significant difference (F-value=16.558, P<0.001). The PLR measure at the baseline and after three months showed a statistically significant difference (F-value=13.966, P<0.001).

## Discussion

This study was conducted at SRM Medical College Hospital and Research Centre between March 2018 and August 2020. A total of 120 subjects were included in the study based on the inclusion and exclusion criteria. These subjects were divided into four groups, with 30 patients in Group 1 which is the control group and 30 each in Groups 2 (patients with normoalbuminuria), 3 (patients with microalbuminuria), and 4 (patients with macroalbuminuria or overt proteinuria). 

In patients with T2DM, microalbuminuria plays an important role in the diagnosis and progression of DN. The cardiovascular mortality associated with microalbuminuria was found to be 2.4 times greater. It is not clearly understood how microalbuminuria increases the risk for ESRD and cardiovascular mortality, but the presence of inflammatory markers and endothelial dysfunction may be responsible for the increased risk in T2DM patients with microalbuminuria. In this study, we aimed to study the relationship between MHR, NLR, and PLR and albuminuria in patients with T2DM.

Age, gender, and proteinuria

In this study, among a total of 120 participants, 54.2% were female, and 45.8% were male. The maximum number of participants is seen in the age group 51-60 years with a mean age of 53±8 years. There was no statistical significance between age, gender, and proteinuria. In this study, 24.2% were smokers and 26.7% consumed alcohol. In comparison with the groups, smoking and alcohol showed no statistical significance with proteinuria (Table 1).

**Table 1:** Demographic data of the study # no statistical difference, P>0.01

Parameters		Groups				X^2 ^value	P-value
		I	II	III	IV		
Age		N=30	N=30	N=30	N=30	13.408	0.145 #
	Up to 40 years	4	1	1	0		
	41-50 years	13	5	9	11		
	51-60 years	11	18	15	14		
	Above 60 years	2	6	5	5		
Gender	Female	10	19	18	18	7.083	0.069 #
	Male	20	11	12	12		
Smoking	Absent	19	24	24	24	3.410	0.333 #
	Present	11	6	6	6		
Alcohol	Absent	22	25	22	19	3.068	0.381 #
	Present	8	5	8	11		

In this study, the mean creatinine value in patients with normoalbuminuric was 1.15±1.78 mg/dL, with microalbuminuria was 0.80±0.19 mg/dL, and with macroalbuminuria was 0.81±0.17 mg/dL, showing no significant association between creatinine and proteinuria. However, eGFR showed a significant difference between the groups. It was found that eGFR was inversely proportional to the grade of proteinuria.

Lian et al. 2021 mentioned that glycated hemoglobin (HbA1c) is an independent factor in increased albuminuria; similarly, in this study, there was a positive correlation between HbA1c values and proteinuria (P<0.001) [25].

Higher serum hsCRP concentration in patients with T2DM may be a risk factor that gives rise to DN. In this study, there was a positive correlation between hsCRP and grade of proteinuria with a mean hsCRP of 0.84±0.59 mg/L.

In recent studies, a positive correlation was found between WBC, neutrophil count, and albuminuria level, whereas there was a negative correlation between WBC and lymphocyte count, which in turn may suggest that albuminuria level is related to the inflammatory response [26]. On comparison of the total count with the groups, it is observed that there is an increase in the total counts with the increase in UACR (P=0.061).

Many studies have found low HDL cholesterol levels to be associated with increased UACR and low GFR in patients with chronic renal failure. A study conducted by Ravid et al. concluded that high serum total cholesterol and low serum HDL cholesterol were risk factors for the progression of DN [27]. In this study, the HDL cholesterol level was significant (P=0.035) with the macroalbuminuria group (mean 41.30±7.19) as compared to the microalbuminuria group (mean 39.63±7.17).

Monocytes and macrophages have a key role in the inflammation process during the development and progression of atherosclerosis [28]. In this study, the monocyte count was found to be higher in the macroalbuminuria group (mean of 530.53±164.79) as compared to the normoalbuminuric and microalbuminuria group (mean of 486.57±158.79) indicating a statistical difference (P<0.001) (Table 2).

**Table 2:** Mean and standard deviations of the blood parameters # no significant difference, * significant at P-value <0.05, ** significant at P-value <0.005, *** significant at P-value <0.0005, eGFR: estimated glomerular filtration rate, HDL: high-density lipoprotein, hsCRP: high-sensitivity C-reactive protein, HbA1c: hemoglobin A1C

Parameters	Groups								F-value	P-value
	I		II		III		IV			
	Mean	SD	Mean	SD	Mean	SD	Mean	SD		
Creatinine (mg/dL)	0.75±0.19		1.15±1.78		0.80±0.19		0.81±0.17		1.228	0.303 #
eGFR (mL/min/1.73m^2^)	102.60±13.24		99.40±12.34		91.00±19.27		88.62±14.64		5.832	0.001**
Hemoglobin (g/dL)	12.56±1.72		10.92±1.63		11.93±1.40		11.89±1.41		5.775	0.001**
Total counts (cells/cu.mm)	6033.33±803.15		6126.67±1038.88		666.67±1337.87		6834.47±1301.27		3.596	0.016*
HDL (mg/dL)	39.40±5.46		36.67±3.96		41.30±7.19		39.63±7.17		2.968	0.035*
Absolute lymphocyte count (cells/liter)	2342.67±330.80		2398.67±432.03		2368.67±574.49		2456.67±532.71		0.317	0.813 #
Absolute neutrophil count (cells/liter)	2693.67±454.94		2835.50±749.21		3130.17±819.02		3344.50±719.21		5.245	0.002**
Absolute monocyte count (cells/liter)	308.30±66.30		332.93±74.62		486.57±158.79		530.53±164.79		23.479	0.0005**
Platelet count (cells/cu.mm)	197200.00±13892.20		207166.67±25460.02		242685.67±61686.38		252484.17±64174.42		9.853	0.0005**
hsCRP (mg/dL)	0.41±0.37		0.84±0.59		1.70±0.77		1.71±0.72		31.775	0.0005**
HbA1c (%)	5.13 ± 0.49		9.24±1.36		8.48±0.95		8.63±1.17		94.652	0.0005**
Urine spot PCR (mg/mmol)			11.06±5.44		88.82±52.59		480.53±128.12		296.542	0.0005***

MHR and proteinuria

Many epidemiological studies have shown dyslipidemia to be an important risk factor for the progression of chronic renal failure. A recent experimental study has shown “that impaired cholesterol efflux pathways and decreased HDL levels stimulate growth in hematopoietic stem cells, especially in the monocyte cell lines, and thereby promote atherosclerotic plaque formation in mice" [29]. In our study, the monocyte count was found to be higher in the macroalbuminuria group as compared to the normoalbuminuric and microalbuminuria groups.

Kanbay et al. determined the ratio between monocyte count and HDL cholesterol levels in the circulation and designated it as MHR. They determined this ratio to be an independent risk factor for chronic renal patients in major cardiovascular events in terms of poor prognosis [30]. In this study, the mean MHR in the four groups was 86.09±13.88, 89.17±19.78, 102.65±9.97, and 105.06±9.62, respectively. The MHR was significantly higher in macroalbuminuria as compared to normoalbuminuric and microalbuminuria (P<0.001).

NLR and proteinuria

Afşar et al. reported that an increased NLR was independently related to UAE [19]. In addition, Akbaş et al. reported that HbA1c and NLR are independent predictors for albuminuria [20]. This study also confirmed similar findings.

PLR and proteinuria

PLR has recently been introduced as a marker of several inflammatory conditions not only in malignancies but also in other pathologies involving the heart, thyroid, kidney, and liver and even DM, metabolic syndrome, and essential hypertension. In this study, the mean PLR in the four groups was 0.41±0.37,0.84±0.59, 1.70±0.77, and 1.71±0.72, respectively, showing a positive correction between PLR and proteinuria (P<0.001) (Table 3).

**Table 3:** Analysis of the MDL and PLR # no significant difference, * significant at P-value <0.05, ** significant at P-value <0.005, *** significant at P-value <0.0005, MDL: monocyte-to-high-density lipoprotein cholesterol ratio, PLR: platelet-to-lymphocyte ratio

Groups	Baseline		F-value	P-value	Third month		F-value	P-value
	Mean	SD			Mean	SD		
MDL								
I	7.88	1.61	16.558	0.0005**	78.13	1.31	18.989	0.0005**
II	9.17	2.26			8.96	1.90		
III	12.13	4.33			12.11	3.87		
IV	13.79	5.14			13.51	4.57		
PLR								
I	86.09	13.88	13.966	0.0005**	87.02	11.58	16.982	0.0005**
II	89.17	19.78			88.28	17.03		
III	102.65	9,97			101.18	8.63		
IV	105.06	9.62			104.72	8.32		

Analysis of the results after three months

In this study, after a follow-up of three months, MHR, NLR, and PLR demonstrated a positive correlation with UACR levels and a statistically significant difference between their baseline and three-month values (P=0.001).

Limitations

One of the limitations of this study was the relatively small sample size. As the study had a short period of follow-up of three months and as the progression of DN is a chronic process, the credibility of the MHR could not be properly assessed. A larger study population and a longer period of follow-up are needed to confirm the findings.

## Conclusions

This is a prospective observational study that included a total of 120 patients with T2DM who fulfilled the inclusion criteria and exclusion criteria. Higher HbA1c and hsCRP values increase the chances of developing DN. In this study, the relationship between the MHR, NLR, PLR, and albuminuria levels in DN patients was analyzed which showed a positive correlation with UACR levels. This supports the thesis that the positive correlation of the MHR, NLR, and PLR with UACR would serve as an inflammatory marker. MHR can be used as an easily accessible and inexpensive prognostic marker in patients with DN. The correlation between the MHR and albuminuria levels showed that the MHR had an early prognostic value in the development of DN in T2DM patients. Based on the clinical results, the one-time measurement of MHR and NLR was clinically significant in predicting the disease state. The difference in the three-month follow-up was done mainly to check the consistency of the results, and further long-term follow-up studies could be done to implicate variations that could happen with treatment.
